# Change in Mesoherbivore Browsing Is Mediated by Elephant and Hillslope Position

**DOI:** 10.1371/journal.pone.0128340

**Published:** 2015-06-17

**Authors:** D. D. Georgette Lagendijk, Maria Thaker, Willem F. de Boer, Bruce R. Page, Herbert H. T. Prins, Rob Slotow

**Affiliations:** 1 School of Life Sciences, University of KwaZulu-Natal, Westville Campus, Private Bag, X54001, Durban, 4000, South Africa; 2 Centre for Ecological Sciences, Indian Institute of Science, Bangalore, 560012, India; 3 Resource Ecology Group, Wageningen University, Droevendaalsesteeg 3a, 6708 PB, Wageningen, The Netherlands; 4 Department of Genetics, Evolution and Environment, University College, London, United Kingdom; Key Laboratory of Tropical Forest Ecology, Xishuangbanna Tropical Botanical Garden, Chinese Academy of Sciences, CHINA

## Abstract

Elephant are considered major drivers of ecosystems, but their effects within small-scale landscape features and on other herbivores still remain unclear. Elephant impact on vegetation has been widely studied in areas where elephant have been present for many years. We therefore examined the combined effect of short-term elephant presence (< 4 years) and hillslope position on tree species assemblages, resource availability, browsing intensity and soil properties. Short-term elephant presence did not affect woody species assemblages, but did affect height distribution, with greater sapling densities in elephant access areas. Overall tree and stem densities were also not affected by elephant. By contrast, slope position affected woody species assemblages, but not height distributions and densities. Variation in species assemblages was statistically best explained by levels of total cations, Zinc, sand and clay. Although elephant and mesoherbivore browsing intensities were unaffected by slope position, we found lower mesoherbivore browsing intensity on crests with high elephant browsing intensity. Thus, elephant appear to indirectly facilitate the survival of saplings, via the displacement of mesoherbivores, providing a window of opportunity for saplings to grow into taller trees. In the short-term, effects of elephant can be minor and in the opposite direction of expectation. In addition, such behavioural displacement promotes recruitment of saplings into larger height classes. The interaction between slope position and elephant effect found here is in contrast with other studies, and illustrates the importance of examining ecosystem complexity as a function of variation in species presence and topography. The absence of a direct effect of elephant on vegetation, but the presence of an effect on mesoherbivore browsing, is relevant for conservation areas especially where both herbivore groups are actively managed.

## Introduction

The African savanna biome, characterised by a high degree of horizontal and vertical spatial heterogeneity, harbours one of the most diverse assemblages of large herbivores [1,2]. As would be expected, species use the landscape differently depending on their specific nutritional requirements and the spatial heterogeneity of the available resources [3]. Many complex factors determine herbivore foraging behaviour and the consequent use of the landscape, including the quality, availability, spatial distribution of resources, and specific nutritional requirements [3,4], as well as predation risk [5] and competition from other herbivores [6]. Therefore, the impact of herbivores on vegetation is spatially heterogeneous across the savanna landscape [7,8].

In savanna ecosystems, much of the spatial heterogeneity of herbivory is understood as a function of the distance to major water sources, such as rivers and waterholes, at the landscape level [9,10]. However, ecohydrological conditions can vary at much smaller spatial scales, such as within hillslopes.

During the wet season, water and sediments are also transported downslope through runoff, where they are captured by the vegetation in lower areas (run-on) and partially stored in the soil [11,12]. This runoff also prolongs the growing seasons in low-lying areas, resulting in a different composition of plant species along slopes [13]. The nutrients that thus accumulate at lower slope positions result in a high quality soil resource base for plant regeneration and growth [11,12]. Consequently, plants at lower slope positions tend to be of a higher nutritional quality and more palatable than plants on crests [14]. The effect of slope position (e.g., footslopes vs. crests) on the foraging behaviour of grazers has been well studied [14,15]. For example, Macandza et al. [15] found that African buffalo *Syncerus caffer* grazing on granite landscapes in Kruger National Park, South Africa, utilised uplands in winter and midslopes in summer, but that bottomlands were only moderately used. Grant and Scholes [14] reported that concentrate feeders, such as impala *Aepyceros melampus* and wildebeest *Connochaetes taurinus* prefer to utilise sodic sites, which are situated on footslopes. However, less is known about the utilisation of hillslopes by browsers, especially in savanna ecosystems. In Botswana, areas with gentle undulating slopes were found to have a higher browsing pressure by African elephant *Loxodonta africana* than flat areas [7]. The gap in knowledge of browsing on slopes in savanna systems is striking, as many African savannas consist of undulating landscapes, with slopes of varying steepness. The savannas in the Greater Kruger Area, where this study is based, are characterised by distinct catenal landscapes, with low to moderate relief and often long slopes [16].

Vegetation dynamics and composition are also strongly influenced by herbivore impacts, of which elephant herbivory and damage are among the strongest drivers of savanna dynamics [8,17]. Most typically, the foraging behaviour of elephant, which includes bark removal and toppling of trees, can negatively affect tree populations [8,17,18]. Notably however, evidence of the negative effects of elephant on savanna vegetation has been obtained in areas with longstanding populations of the megaherbivore (e.g., > 100 years in Kruger National Park), and little is known about the immediate and short-term effects of elephant in areas where they have been (re)introduced. Mesoherbivores (medium-size herbivores between 50–450 kg; [19,20]) have also shown to negatively affect regeneration of woody vegetation [18,21–24]. For example, browsing by nyala *Tragelaphus angasii* was found to inhibit seedling recruitment and affect tree species composition in Sand Forest, South Africa [23,24]. Elephant and mesoherbivores are likely to interact, and impact vegetation composition in different ways. Elephant can facilitate foraging for mesoherbivores by increasing browse availability at lower levels after impacting large trees, by breaking of branches or inducing a coppicing response from the tree [25–27]. On the other hand, interference competition occurs between elephant and other herbivores at waterholes [28], and elephant are known to displace meso- and smaller-sized herbivores in Sand Forest [24]. Whether spatial segregation, competition or facilitation between elephant and mesoherbivores influence their impacts on vegetation at the hillslope scale and in savanna ecosystems is currently unknown.

We aimed to determine the combined effect of short-term elephant presence (< 4 years) and hillslope position on tree species assemblages, resource availability, browsing intensity and soil properties. Using an experimental approach, we thus examined woody plant communities at the hillslope scale, and the effect and interaction of herbivory by elephant and mesoherbivores to understand differences in vegetation composition and structure. We also examined the effect of soil properties on woody species assemblages on footslopes and crests, as soil properties may differ between slope positions, and are an important determinant of species composition [13]. Specifically, we tested whether tree species assemblages differ between slope positions, and in response to differences in soil nutrient and browsing levels. Given that nutrient and moisture levels were expected to be higher on footslopes than on crests of hills, we also predict higher tree densities with a concomitant increase in browsing intensity on footslopes as plants should be more nutritious and palatable at this landscape position, resulting in dissimilar height class distributions of the woody species. We further expect a facilitating effect of elephant browsing on herbivory by mesoherbivores in line with previous findings [25–27], as they are known to make more browse available at lower height levels.

## Methods

### Study area

We conducted this study in Balule Nature Reserve (hereafter Balule; 350 km^2^) which borders Kruger National Park in Limpopo Province, South Africa (24°21’- 24°17’ S; 31°01’- 30°95’ E). The open savanna woodland is dominated by *Combretum apiculatum*, *Grewia* spp., *Senegalia nigrescens* and *Sclerocarya birrea*. The fuel load in the study area is low and fires rarely occur (M. Cesare, pers. comm.). Balule has a moderate undulating topography throughout with elevations ranging from 340–450 m above sea level. The underlying substrate is granite with soapstone outcrops, nutrient-poor shallow orthic soils with quartz gravel higher up the slopes, and rich red soils in the lower lying areas ([29]; M. Cesare, pers. comm.). The climate is sub-tropical with hot, wet summers (November-April) and cool, dry winters (May-October). Temperatures range from 4–40°C, with a mean annual rainfall of 401 mm (1985–2007; [30]).

Fences between Balule and neighbouring reserves were removed in May 2005, after which elephant moved into Balule, providing the opportunity to study the relatively short-term effect of elephant presence (< 4 years) on vegetation and other herbivores. Before 2005, only seven elephant were present in Balule (M. Cesare, pers. comm.), and the effect of such a low elephant density (i.e., 0.02 elephant km^-2^) would presumably have been minimal. The historically low elephant impact in Balule is further supported by the fact that elephant were locally extinct until 1903 [31]. Part of this study was conducted in a small part of Balule, namely within Ukhozi Nature Reserve (hereafter Ukhozi; 20 km^2^). Between 1987 and 2007, Ukhozi was used as a breeding farm for African buffalo, for which 15 km^2^ were fenced off (C. Ferguson, pers. comm.). The Balule areas bordering Ukhozi were, at that time, used as private and tourist game viewing areas. In November 2007, fences were replaced with an electric elephant exclusion fence, allowing all wildlife, except giraffe *Giraffa camelopardalis* and elephant, to move freely between Balule and Ukhozi. Giraffe density in Ukhozi was 0.3 km^-2^ (Balule 0.8 km^-2^) and elephant density in Balule was 1.2 km^-2^. Other browsing ungulates in Ukhozi include kudu *Tragelaphus strepsiceros* (0.4 km^-2^; Balule 1.5 km^-2^), impala (10 km^-2^; Balule 13 km^-2^) and bushbuck *Tragelaphus scriptus* (0.3 km^-2^; Balule no count available). Densities were derived from helicopter game counts in 2007.

Permission to conduct this research in Balule was obtained from C. Ferguson, the warden of the reserve.

### Experimental design

**Fig 1:**
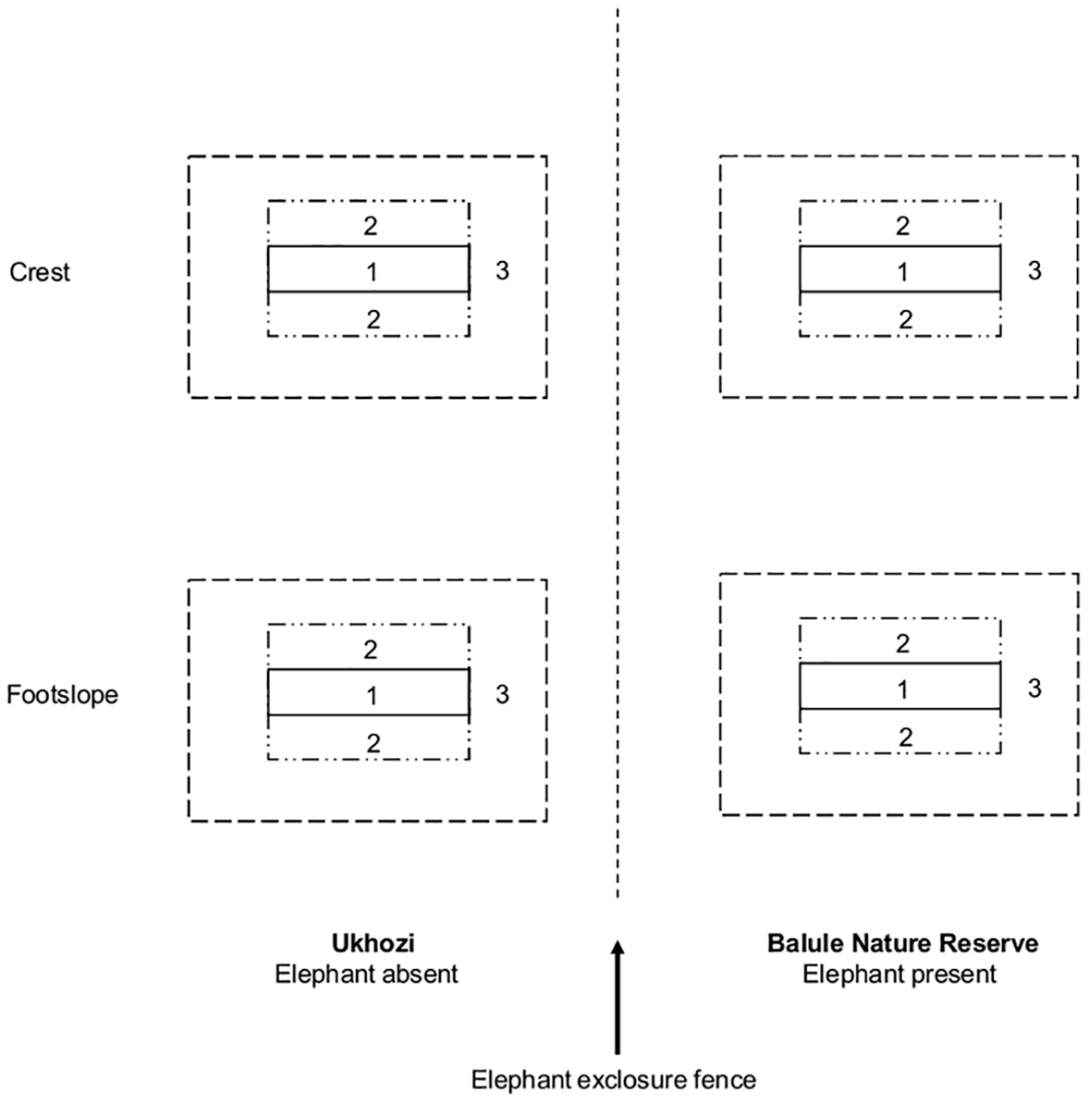
Schematic overview of the four treatments (slope position: footslope vs. crest; elephant: absent vs. present) as laid out on each of the five sites. Vegetation within each treatment was recorded using three quadrats: (1) seedlings only: ≤ 0.5 m; (2) woody individuals above 0.5 m; (3) species not encountered in either quadrat 1 or 2 (all height classes included).

Five crests (range: 420–450 m asl) and footslope (range: 405–435 m asl) sites were selected on the border of Ukhozi (i.e., the 15 km^2^ elephant free zone) and the rest of Balule. Due to the relatively small size of the elephant free zone of Ukhozi, the distances between sites ranged from a minimum of 0.8 km to a maximum of 6.0 km. Each of the five sites consisted of four plots (Fig 1); two paired on each reserve (elephant effect: elephant presence vs. absence), and each of these two positioned on either a crest or a footslope (slope position effect). Bottomlands, where the largest effect was expected, were too narrow in size to sample, and we therefore selected the footslope. Our sampling design resulted in four treatments: (1) crest without elephant; (2) crest with elephant (3); footslope without elephant; (4) footslope with elephant (N = 5 for each treatment), where the elephant effect was thus paired per slope position.

### Woody vegetation sampling

The woody vegetation was sampled in December 2008. Three quadrats were placed in each plot (Fig 1). Quadrat 1 (2 x 40 m), where only seedlings (≤ 0.5 m) were recorded, was nested within quadrat 2 (30 x 40 m), where individuals > 0.5 m were recorded, which was nested within quadrat 3 (50 x 100 m), where individuals of additional species, i.e., species not encountered in either quadrat (1) or (2) were recorded, including all height classes. This sampling design is similar to Kalwij et al. [10]. In each quadrat, all woody individuals were counted, identified to species, and tree height of the individual was recorded. The heights of trees ≥ 2 m were estimated using the height of an observer as a scale following Shannon et al. [32]. Plant names follow Coates Palgrave [33] and Kyalangalilwa et al. [34].

### Soil properties

Soil samples in relation to slope position were collected in May 2010. A composite soil sample of 500 g fresh weight (consisting of 10 random samples lumped per plot) was taken from the top 10 cm of the soil layer (cf. [35]). Litter and stones larger than 1 cm in diameter were removed upon sampling. Samples were air dried prior to transportation. The following properties were analysed from the soil samples at the Soil Fertility and Analytical Service Section of the Agricultural Research Council (ARC) in Cedara, South Africa: Phosphorus (P), Potassium (K), Total Nitrogen (N), Copper (Cu), Calcium (Ca), Magnesium (Mg), Manganese (Mn), Zinc (Zn), pH, total cations and texture (% silt, sand and clay), following Boyes et al. [36].

### Browsing intensity

Browsing events on trees by mesoherbivores (i.e., impala or bushbuck) and elephant were recorded once for each woody plant in each quadrat during the vegetation sampling in December 2008. Each woody plant was carefully examined for browsing damage, following Lagendijk et al. [23]. Leaf stripping and branch removal by even young elephant is easily detected on the plant, as well as single bites by mesoherbivores. Only accumulated browsing events (i.e., total of all events, regardless of size of bite) positively ascribed to either elephant or mesoherbivores were included in the analyses.

### Data-analyses

#### Differences in woody vegetation properties

Densities of individuals and stems of woody plants were expressed per 1 ha for comparison. We analysed species richness (total number of species) and tree species assemblages (which includes both composition and abundance of species) in order to determine if vegetation communities differed between footslopes and crests, and with or without elephant. Differences in species richness were tested using a nested ANOVA design, in which slope position was nested in site and elephant presence nested in slope position. A two-way crossed analysis of similarity (ANOSIM) was used to test for differences in species assemblages between slope position and elephant effect [37]. Species abundances were fourth-root transformed, which reduces the influence of the more abundant species in the calculation of the Bray-Curtis dissimilarities [37]. ANOSIM calculates the R statistic which ranges between 0–1; the closer a significant R-value is to one, the more distinct the species assemblages [37].

Two possible demographic responses to browsing can be expected, namely increased mortality or enhanced coppicing. Therefore, we investigated changes in both the density of individuals (which measures mortality, but also reflects recruitment), and stem density (which measures the coppicing response and the mortality of stems). Nested ANOVAs, wherein slope position was nested in site and elephant presence was nested in slope position, were used to test whether footslopes have higher densities of trees and stems, and whether this was affected by elephant presence. Log_10_-transformations were applied to stem densities to satisfy assumptions of normality and equality of variance.

To prevent species characteristics and species assemblages from obscuring the analyses of the structural distributions of trees and browsing intensity, the following analyses were restricted only to woody species occurring in all four treatments. *Grewia* spp. and *Ozoroa* spp. were pooled within genus due to hybridisation within each genus. Consequently 19 species (i.e., totalling to 97% of all trees) were included in the analyses (S1 Table).

Since height and diameter of trees are correlated, we only analysed the effects of slope position and elephant presence on one of these variables, namely height. Trees were allocated to five functional height classes (≤ 0.5 m: seedling; 0.51–1.5 m: sapling; 1.51–3 m: small tree; 3.01–5 m: medium tree; > 5.01 m: tall tree [10,32,38,39]). These height classes roughly correspond to the limits at which browsing impact by different-sized herbivores occurs [23].

The effects of slope position, elephant presence and site on the densities of each functional height class of the 19 tree species were analysed using a nested MANOVA. To satisfy assumptions of normality and equality of variances, we applied log_10_-transformations on saplings and small trees, and square-root transformation on large trees.

#### Differences in soil properties

A nested MANOVA was used to test if soils had higher nutrient concentrations on footslopes than on crests (with slope position nested in site; elephant presence was not included as a factor because the short exposure to elephant would not be expected to influence soil properties). The soil properties P, K, Total N, Cu, Mg, Mn, Zn, pH, total cations, sand and clay were included in the model after arcsine-transformation (except for pH and total cations). Since sand, silt and clay make up 100% of the soil texture, only two of these variables were included in the model, namely sand and clay. Soil parameters were tested for correlation using Spearman rank correlations and omitted when *r*_*s*_ ≥ 0.95 [37]. Ca was, subsequently, omitted from the analyses due to high correlation with total cations (*r*_*s*_ = 0.985, *P* < 0.001).

#### Relationship between species assemblages and soil parameters

The relationship between species assemblages and soil parameters was investigated using the BIO-ENV procedure in PRIMER [37]. Of the soil texture properties, sand and clay were again included in the analysis. All soil parameters were arcsine-transformed with the exception of pH and total cations. Ca was again omitted due to high correlation with total cations. In order to match the species assemblage data to the soil parameters, a similarity matrix of the latter based on normalised Euclidean distance was used, which was linked to the species similarity matrix. During the BIO-ENV procedure, the parameters maximising the rank correlation (*r*_*s*_) between the two matrices are selected, and thus provide the best match for explaining the variation in species assemblages.

#### Differences in browsing intensity

To determine if browsing intensity was higher at footslopes than at crests, or affected by elephant access, we analysed browsing intensity by mesoherbivores using a nested ANOVA. Browsing intensity by mesoherbivores on the 19 species was calculated as the number of browsing events (i.e., impacted trees) per hectare. Elephant browsing intensity on these species was calculated similarly, and, using ANOVA, we tested if elephant browsing intensity was higher at footslopes than at crests.

We used a linear regression to determine if elephant browsing affected herbivory by mesoherbivores. Elephant browsing was measured as browsing events (i.e., impacted trees) per hectare. To control for among-site effects (i.e., local non-elephant density), browsing events by mesoherbivores in Balule (elephant present) were subtracted by their browsing events on Ukhozi (elephant absent) for each pair of corresponding elephant-effect plots, which gives us the relative browsing response by mesoherbivores to elephant browsing.

ANOSIM and BIO-ENV were analysed in PRIMER 6 (PRIMER-E Ltd.). All other statistical tests were performed using SPSS 15.0 (SPSS Inc., Chicago, USA).

## Results

In total, 4661 individual trees of 68 woody species were recorded in the study (S2 Table). We found no effect of site on species richness (*P* = 0.34), density of functional height classes (*P* = 0.16), soil properties (*P* = 0.17), and browsing intensity by mesoherbivores (*P* = 0.46). These variables, among others, however, were strongly affected by slope position, elephant presence, and the indirect effect of elephant on mesoherbivores (see below).

### Effects of slope

Species richness was higher on footslopes than on crests, irrespective of elephant presence (*F*_*4*,*8*_ = 5.566, *P* = 0.02; S2 Table). Footslopes harboured 20 species not encountered on crests, and crests had 13 species that were not recorded on footslopes, consequently both slope positions had 35 species in common. Concordant with this, ANOSIM indicated that woody species assemblages on footslopes differed significantly from assemblages on crests (ANOSIM: *R* = 0.318, *P* = 0.004).

**Fig 2:**
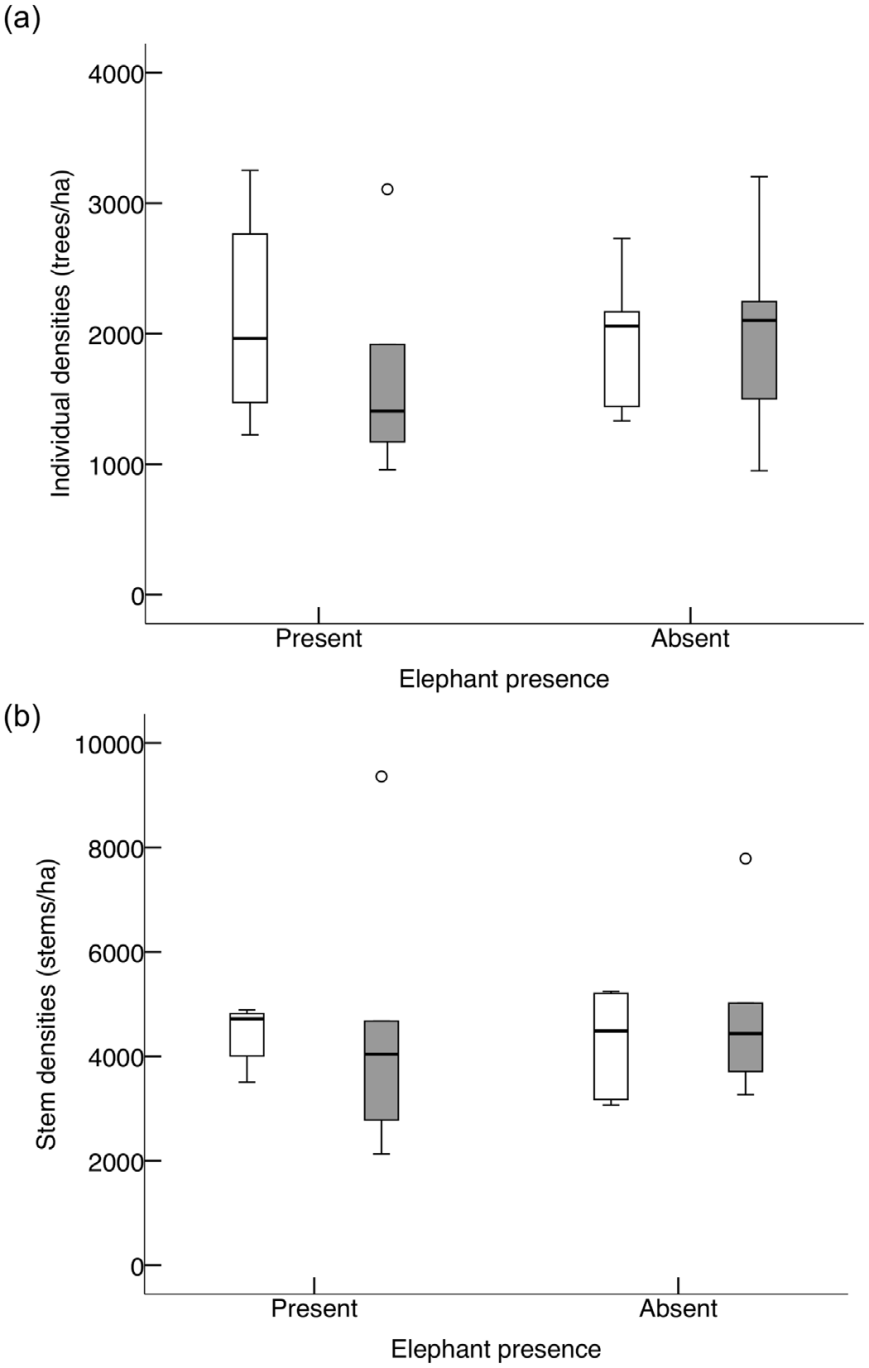
Densities of all woody species in areas with elephant presence and absence per slope position (crests: white bars; footslopes: grey bars): (a) individual densities (trees/ha); (b) stem densities (stems/ha, which includes coppicing effects). Shown are range (whiskers), 25 and 75% quartiles (box), median (line), and circles are outlying values. N = 5 replicates per treatment.

**Fig 3:**
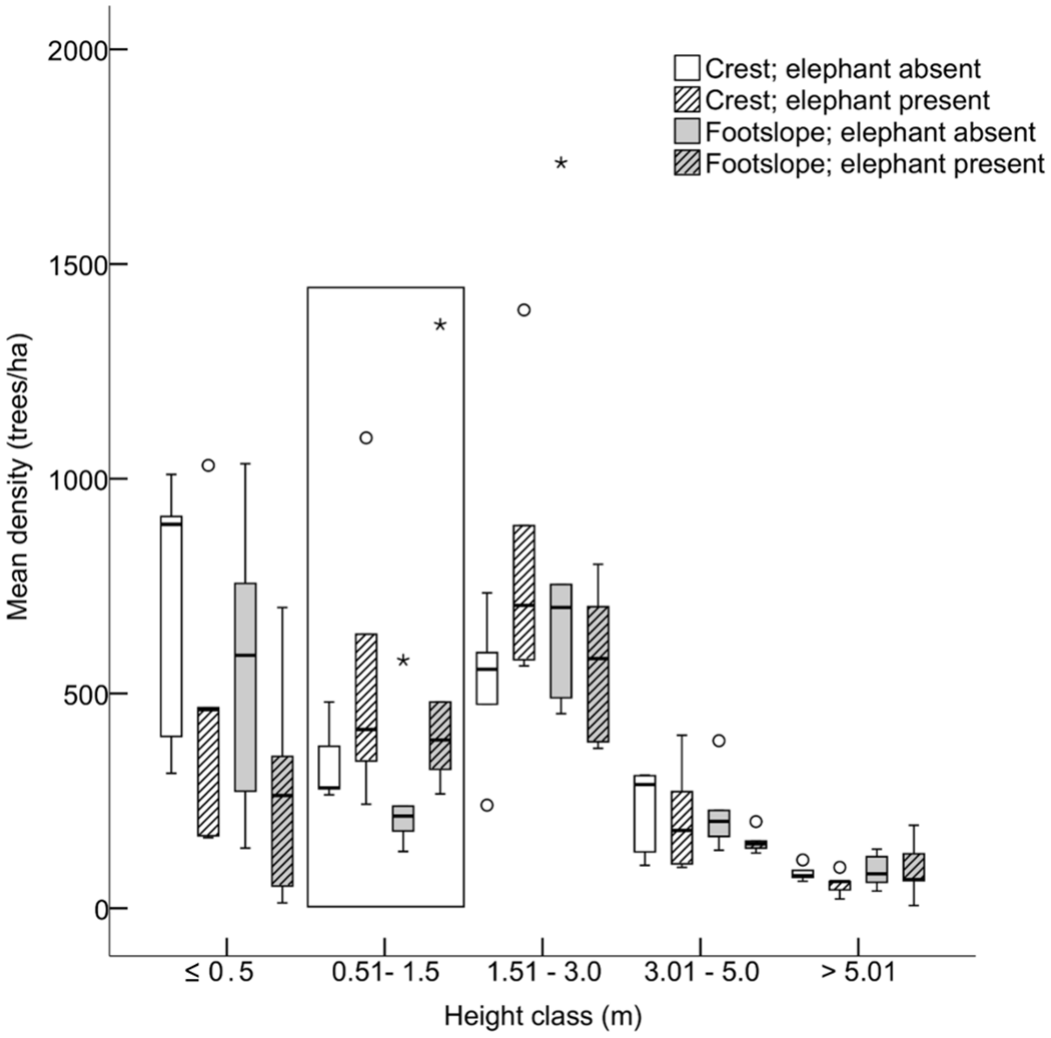
Mean density of trees across the height classes for the 19 species combined per treatment. Note the higher sapling densities in presence of elephant. Elephant presence and slope position had no significant effect on densities in any of the other height classes. Data are range (whiskers), 25 and 75% quartiles (box), median (line), stars and circles are extreme and outlying values, respectively. N = 5 replicates per combination of slope position and elephant presence.

Contrary to our expectation, overall tree and stem densities were not significantly higher on footslopes than on crests (tree densities: *P* = 0.21; stem densities: *P* = 0.32; Fig 2 and S3 Table). Given that tree and stem densities were not significantly different between slope positions, there was no apparent coppicing response. Furthermore, there was no significant effect of slope position on densities of functional height classes (restricted to 19 species occurring in all four treatments; slope position: Pillai’s trace: *F*_*20*,*28*_ = 1.162, *P* = 0.35; Fig 3 and S4 Table).

Slope position significantly affected multiple soil properties (Pillai’s trace: *F*_*50*,*25*_ = 2.317, *P* = 0.013; S5 Table). Footslopes had significantly higher concentrations of P (*F*_*5*,*10*_ = 3.401, *P* = 0.047), K (*F*_*5*,*10*_ = 5.310, *P* = 0.012), Mg (*F*_*5*,*10*_ = 7.565, *P* = 0.004), and a higher total cations (total cations ~ to Ca: *F*_*5*,*10*_ = 8.684, *P* = 0.002), but crests had higher concentrations of Cu (*F*_*5*,*10*_ = 14.200, *P* < 0.0001). Slope position did not have a significant effect on concentrations of Mn (*P* = 0.087), Zn (*P* = 0.734), total N (*P* = 0.474), pH (P = 0.070), sand (*P* = 0.799) and clay (*P* = 0.593). Variation in woody species assemblages between slope positions was statistically best explained by total cations (collinear with Calcium), Zn, sand (%) and clay (%) (BIO-ENV: *ρ*_*s*_ = 0.374). Species associated with high concentrations of these soil properties included *Balanites maughamii*, *Manilkara mochisia*, *Pappea capensis* and *Senegalia senegal*.

**Fig 4:**
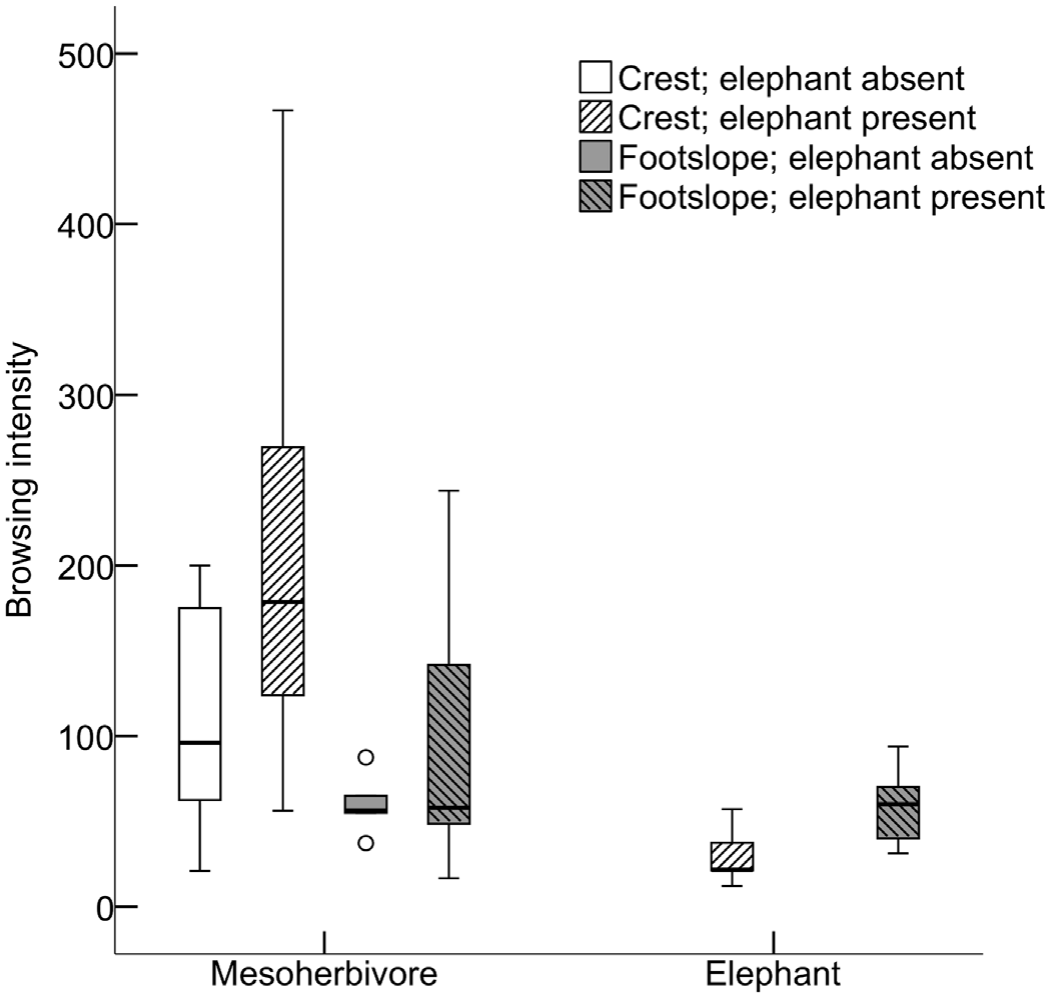
No significant differences in browsing intensity (number of browsing events per hectare) by mesoherbivores and elephant on the 19 plant species in the different treatments. Data are range (whiskers), 25 and 75% quartiles (box), median (line), and circles are outlying values. N = 5 for each treatment.

Browsing intensity of elephant on the 19 woody species (*P* = 0.065) as well as the browsing intensity of mesoherbivores (*P* = 0.213) were not significantly affected by slope position (Fig 4 and S6 Table).

### Effects of elephant

Species richness (*P* = 0.42) and species assemblages (ANOSIM: *R* = 0.004, *P* = 0.48) were similar in areas with and without short-term elephant presence. Similarly, overall tree densities (i.e., including all height classes and all species) and stem densities were similar in areas with and without elephant presence (tree densities: *P* = 0.68; stem densities: *P* = 0.70; Fig 2 and S3 Table), suggesting no apparent coppicing response to elephant presence.

The densities of the different tree height classes (restricted to 19 species occurring in all four treatments), however, showed a significant effect of elephant presence (Pillai’s trace: *F*_*10*,*10*_ = 3.009, *P* = 0.048; Fig 3 and S4 Table). In particular, we found higher sapling (0.51–1.5 m) densities in the presence of elephant (*F*_*2*,*8*_ = 6.499, *P* = 0.021), suggesting displacement of mesoherbivores by elephant (see below).

### Indirect effects of elephant on mesoherbivores

**Fig 5:**
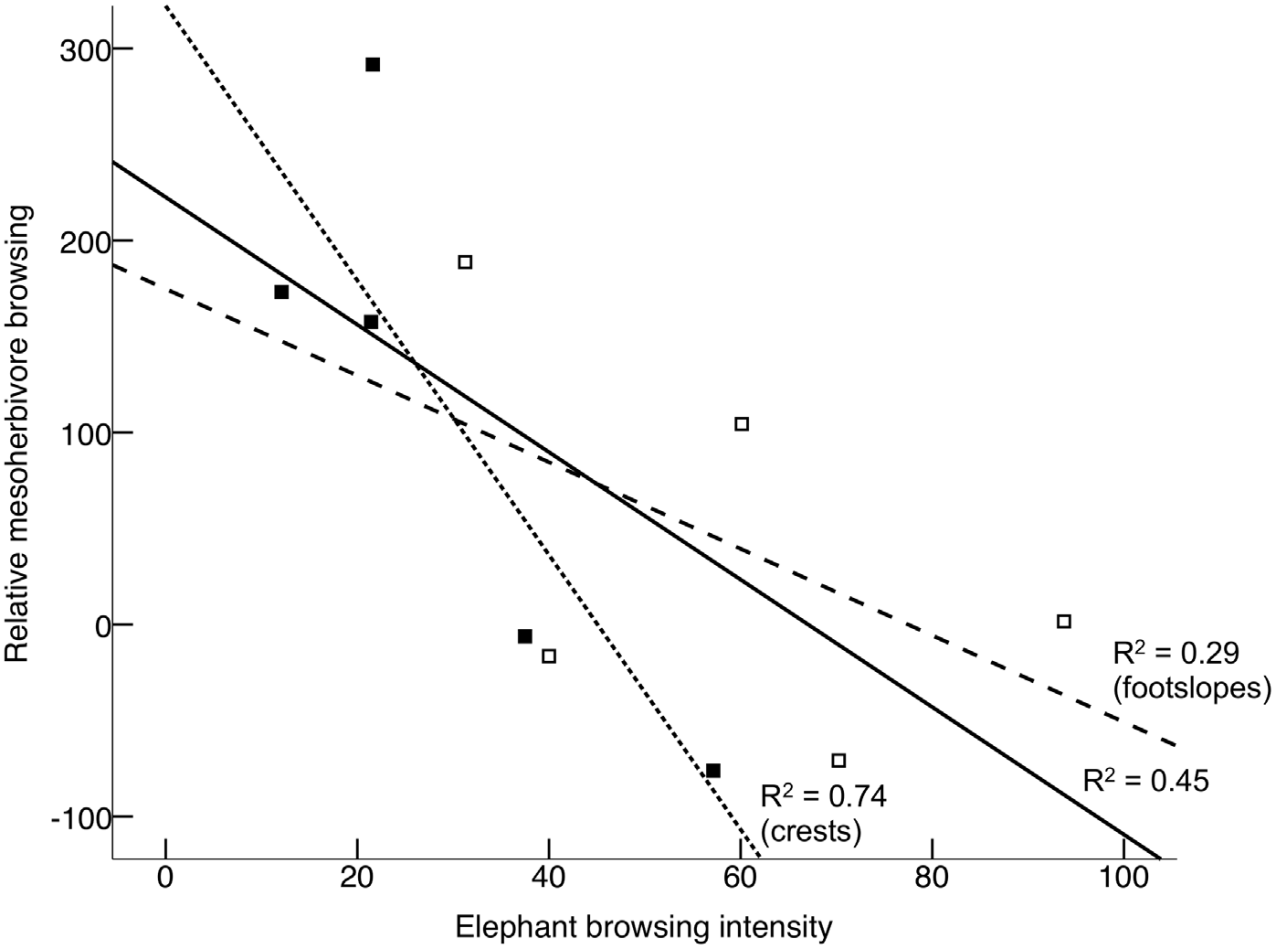
The negative effect of higher elephant browsing on herbivory by mesoherbivores across all 10 plots in the elephant access area (all squares, solid line) on footslopes (open squares, dashed line) and on crests (filled squares, dotted line) (N = 5 sites for each slope position). Elephant browsing was measured as browsing intensity (number of browsing events per hectare). In order to account for among-site effects (i.e., local non-elephant density), relative browsing by mesoherbivores was calculated as the difference in browsing events between Balule (elephant present) and Ukhozi (elephant absent) for each pair of corresponding elephant-effect plots.

Although browsing intensity of mesoherbivores was not significantly affected by the presence of elephant (*P* = 0.191; Fig 4 and S6 Table), browsing intensity by mesoherbivores in Balule, where elephant and mesoherbivores overlap, was significantly lower in areas where elephant browsing intensity was higher (Fig 5; *F*_*1*,*8*_ = 6.661, *P*_(1-tailed)_ = 0.017, *R* ^*2*^ = 0.454). When separated between slope positions, this negative relationship was only significant on crests (Fig 5; *F*_*1*,*3*_ = 8.327, *P*_(1-tailed)_ = 0.032, *R* ^*2*^ = 0.735).

## Discussion

The Greater Kruger Area, in which our study site is situated, is the focus of a national debate on whether elephant should be “lethally managed” or not, mainly because of the prevailing opinion that elephant foraging behaviour negatively impacts on vegetation dynamics [8,40]. Many private land owners and reserve managers are thus reluctant to (re)introduce elephant by removing fences between reserves, despite the fact that elephant are a naturally-occurring herbivore in Southern Africa. Hence, our study focuses on determining the effects of elephant entering an area from which they have been basically absent for a century, and we show that the short-term presence (i.e., < 4 years) of elephant did not have a detrimental effect on vegetation. Recent elephant access to Balule did not affect species richness, woody species assemblages or overall tree and stem densities. However, elephant affected recruitment and regeneration by facilitating the survival of saplings (i.e., 0.51–1.5 m in height) through the displacement of mesoherbivores, which feed on individuals within this height class. We also found that browsing by mesoherbivores was lower in areas with higher elephant browsing.

In terms of small-scale landscape features, slope position was an important feature that affected woody species richness and woody species assemblages. Recruitment and regeneration occurred on both slope positions. Noticeably, slope position did not affect densities of any of the functional height classes (including the recruitment phase), or influence browsing intensity. No site effects were found, which indicates that other landscape-scale features, such as distance to the river or waterholes, did not affect these results.

### Effects of slope position

In accordance with our expectation, slope position affected woody species assemblages (similar to Levick & Rogers [41]). The differences in species assemblages between slope positions in our study area were statistically best explained by total cations, Zinc, and sand and clay (%) content. Species associated with higher soil concentrations of these nutrients were mainly less abundant species (e.g., *Balanites maughamii*, *Manilkara mochisia*, *Pappea capensis* and *Senegalia senegal*). Total cations was highly correlated with Calcium and it may therefore be Calcium (and not total cations), Zinc, and sand and clay content that are important in explaining the variation in species assemblages between the two slope positions. Sand and clay content are proxies for ecological factors such as water holding capacity and soil fertility, while total cations is a proxy for soil fertility as well. Calcium and Zinc are essential elements for plants. Thus these soil properties generally play an important role in plant growth and survival, and could therefore explain the differences in species assemblages between the two slope positions.

Contrary to what we expected, slope position did not affect tree height distributions (including the recruitment phase), or direct browsing intensity. Rainfall and the spatial arrangements of vegetation patches on the hillslope affect the volume of water reaching the lower slope areas [11,42], which determines the increase in moisture and soil quality, and consequently the vegetation community (i.e., assemblages and structural characteristics). However, lower lying areas (i.e., bottomland or footslope) on a slope gradient are not always wetter or higher in all soil nutrients than crests (e.g., [42] for a tropical forest in Panama). For instance, Khomo et al. [43] found that the clay distribution along the catena gradient in Kruger National Park is affected by rainfall, with the least redistribution of clay occurring at drier sites. Our study site is also situated in a low rainfall area, where relatively little redistribution of clay and other nutrients are expected to occur along a hillslope. Subtle differences in nutrient concentrations or texture may favour the occurrence of certain plant species, but may not significantly affect tree height distributions and browsing intensity. In addition, vegetation along the slope can effectively absorb the available run-off [44], by which infiltration mainly occurs higher up the slope. This appears to be the case within the Balule Nature Reserve, as evidenced by the absence of higher tree, stem and recruitment densities, and lack of higher browsing levels on the footslopes. Therefore, trees at the lower part of the slope do not necessarily have enhanced growth or experience increased browsing levels.

The lack of differential elephant browsing intensity between footslopes and crests could be due to similar tree densities on both slope positions, or the circadian movement pattern of elephant over the catenal gradient [45]. Elephant use the lower lying areas during midday when temperatures are high, but typically utilise crests during the night [45]. Thus, foraging impact can be spatially homogenous over the catena, as elephant forage up to 18 h daily [17,46]. Environmental temperature [45,47], and not resource heterogeneity, may thus drive the behavioural decisions and movements of elephant at the small spatial scale of hillslopes.

Despite the absence of significant differences in elephant browsing intensity between slope positions, there was a strong negative relationship between elephant browsing intensity and herbivory by mesoherbivores on crests, but less so on footslopes, suggesting that elephant and mesoherbivores competed for browse resources (see below). This may indicate a strong negative response to the available resources by mesoherbivores on crests. Crests harbour a different species assemblage and lower species diversity compared to footslopes, and thus are likely to be more uniform in nutritional quality than footslopes. Our results add to the contrast of two recent studies, carried out at a larger landscape scale, focussing on herbivory effects at different productivity scales. Asner et al. [48] found herbivore impact to be greater in high nutrient, lowland areas, than in upland areas in Kruger National Park, in close proximity to our study area. However, Pringle et al. [49] found the opposite pattern in Kenya, where higher foraging was recorded on low productivity sites. We also found that soil properties differed between slope positions but these did not influence herbivory between slope positions within our study area. Such variation in herbivory responses across studies further illustrates the complexity of ecosystems and the dubious nature of generalising between different areas.

### Effects of elephant

Upon reintroduction in 2007, elephant densities in Balule were 1.2 elephant km^-2^, which is relatively high compared to elephant densities in other protected areas within South Africa (range: 0.01–1.88 km^-2^; [50]). Therefore, elephant are expected to have a strong effect on vegetation. However, no effect of short-term elephant access (i.e., < 4 years) was detected on species richness, woody species assemblages, and overall densities of trees and stems in Balule. In contrast, vegetation changes both from long and short-term elephant exclusion have been seen in a range of areas in Africa [17,24,38,41]. Although both slope position and elephant access per se do not have an effect on browsing by mesoherbivores, browsing intensity by mesoherbivores is lower at higher elephant browsing levels. Assuming that elephant spend more time in areas where their browsing intensity is higher, the reduced browsing by mesoherbivores can be interpreted either as behavioural displacement of mesoherbivores by elephant or as competition. Competition implies that facilitation of elephant on mesoherbivore browsing, through increased forage availability and quality, does not occur. However, although we do not have behavioural data to support facilitation by elephant, visual observations on trees in the field suggests that elephant did indeed increase browse availability at lower levels by breaking branches. The possibility of behavioural displacement, however, is furthermore substantiated by the fact that there were higher densities of saplings (i.e., 0.51–1.5 m in height), which are within the feeding height range of mesoherbivores (see [51,52]), where elephant were present. Fire is not a driver of these observed differences in vegetation structure, as fire is near to absent from the area due to low fuel loads. Although elephant can thus facilitate mesoherbivores [25–27], we now also propose that elephant browsing activity can displace mesoherbivores, similar to the findings of Lagendijk et al. [24] for elephant displacing nyala in Sand Forest. Elephant access to an area can thereby have a positive effect on sapling regeneration into taller size classes, and as such influence vegetation dynamics.

## Conclusions and Implications

Observed patterns in vegetation composition and herbivory between slope positions are complex to understand due to the numerous interactions present between biotic and abiotic elements. We found differences in some soil properties (e.g., Phosphorus, Potassium, Magnesium, Copper, total cations and Calcium) between crests and footslopes, but apart from soil properties, many other environmental variables such as hydrology, wind exposure and radiation could also contribute to the underlying mechanisms explaining differences in species assemblages, woody vegetation structure and herbivory between slope positions (e.g., [53]). The contrasting results found by other studies (Daws et al. [42] tropical forest; Asner et al. [48] and Pringle et al. [49] for savanna) show that the observed relationships between the spatial pattern on the hillslope scale and the ecological processes do not always hold. In addition to the spatial heterogeneity at regional landscapes, the variation in patterns found across studies emphasises the importance of acknowledging the local topography, and highlights the need for more site-specific management.

The absence of a direct elephant effect on woody species composition, tree densities and height structure is important to recognise, especially within the current ‘elephant debate’ in which conservation managers are concerned with the effects of increasing elephant populations on the sustainability of protected areas [8]. Our observed lack of a direct effect of elephant browsing, as well as the facilitating effect for sapling recruitment, is in contrast to many other studies. Published studies so far typically report negative effects (see [8]), thus creating a unidirectional bias in our understanding of elephant effects at all spatial and temporal scales. It is thus imperative for elephant management that these non-significant as well as positive effects also get reported. Certainly, understanding the scale of elephant impacts is important; while there may not be a direct effect at the local hillslope scale in our study, there may be effects at larger landscape or temporal scales, especially under higher elephant densities.

## Supporting Information

S1 Table List of 19 species included in the functional height class and browsing intensity analyses. Plant names follow Coates Palgrave [32] and Kyalangalilwa et al. [33].
(DOCX)

S2 Table Species composition (trees per hectare) per slope position in areas with and without elephant. (XLSX)

S3 Table Individual tree and stem densities (trees per hectare) per slope position in areas with and without elephant. (DOCX)

S4 Table Tree density (per hectare) across the functional height classes for the 19 species combined per treatment (slope position and elephant presence/absence). (DOCX)

S5 Table Soil properties per slope position in areas with and without elephant. (DOCX)

S6 Table Browsing intensity (number of browsing events per hectare) for mesoherbivores and elephant per treatment (slope position and elephant presence/absence). (DOCX)
