# Supplementary material for: Change in Mesoherbivore Browsing Is Mediated by Elephant and Hillslope Position
Source: PLoS One. 2015 Jun 17;10(6):e0128340. doi: 10.1371/journal.pone.0128340 (PMC4471177; doi:10.1371/journal.pone.0128340)
Supplement: S1 Table — Plant names follow Coates Palgrave [32] and Kyalangalilwa et al. [33]. (DOCX) [file pone.0128340.s001.docx]

S1 Table. List of 19 species included in the functional height class and browsing intensity analyses. Plant names follow Coates Palgrave [32] and Kyalangalilwa et al. [33].

| *Boscia albitrunca* (Burch.) Gilg & Gilg-Ben. | *Ozoroa* spp. |
| --- | --- |
| *Cassia abbreviata* Oliv. | *Peltophorum africanum* Sond. |
| *Combretum apiculatum* Sond. | *Sclerocarya birrea* (A.Rich.) Hochst. |
| *Combretum mossambicense* (Klotzsch) Engl. | *Senegalia erubescens* (Welw. ex Oliv.) Kyal. & Boatwr. |
| *Commiphora africana* (A.Rich.) Engl. | *Senegalia nigrescens* (Oliv.) P.J.H. Hurter |
| *Commiphora mollis* (Oliv.) Engl. | *Sterculia rogersii* N.E.Br. |
| *Dichrostachys cinerea* (L.) Wight & Arn. | *Terminalia prunioides* M.A. Lawson |
| *Grewia* spp. | *Ximenia americana* L. |
| *Jasminum multipartitum* Hochst. | *Ziziphus mucronata* Willd. |
| *Lannea schweinfurthii* (Engl.) Engl. |  |
