# Supplementary material for: Change in Mesoherbivore Browsing Is Mediated by Elephant and Hillslope Position
Source: PLoS One. 2015 Jun 17;10(6):e0128340. doi: 10.1371/journal.pone.0128340 (PMC4471177; doi:10.1371/journal.pone.0128340)
Supplement: S3 Table — (DOCX) [file pone.0128340.s003.docx]

S3 Table. Individual tree and stem densities (trees per hectare) per slope position in areas with and without elephant

| **Site number** | **Slope position** | **Elephant** | **Individual tree densities** | **Stem densities** |
| --- | --- | --- | --- | --- |
| 1 | crest | absent | 2058 | 5244 |
| 1 | crest | present | 1225 | 4006 |
| 2 | crest | absent | 1442 | 3173 |
| 2 | crest | present | 3251 | 4718 |
| 3 | crest | absent | 2730 | 5204 |
| 3 | crest | present | 1963 | 4816 |
| 4 | crest | absent | 2167 | 4485 |
| 4 | crest | present | 2763 | 4888 |
| 5 | crest | absent | 1331 | 3065 |
| 5 | crest | present | 1473 | 3503 |
| 1 | footslope | absent | 2245 | 3710 |
| 1 | footslope | present | 1917 | 4674 |
| 2 | footslope | absent | 2102 | 4435 |
| 2 | footslope | present | 1171 | 2129 |
| 3 | footslope | absent | 3203 | 7787 |
| 3 | footslope | present | 3107 | 9360 |
| 4 | footslope | absent | 1501 | 5017 |
| 4 | footslope | present | 957 | 2779 |
| 5 | footslope | absent | 949 | 3264 |
| 5 | footslope | present | 1406 | 4041 |
