# Supplementary material for: Change in Mesoherbivore Browsing Is Mediated by Elephant and Hillslope Position
Source: PLoS One. 2015 Jun 17;10(6):e0128340. doi: 10.1371/journal.pone.0128340 (PMC4471177; doi:10.1371/journal.pone.0128340)
Supplement: S4 Table — (DOCX) [file pone.0128340.s004.docx]

S4 Table. Tree density (per hectare) across the functional height classes for the 19 species combined per treatment (slope position and elephant presence/absence)

| **Site number** | **Slope position** | **Elephant** | **Seedling** | **Sapling** | **Small tree** | **Medium tree** | **Large tree** |
| --- | --- | --- | --- | --- | --- | --- | --- |
| 1 | crest | absent | 894 | 334 | 578 | 139 | 100 |
| 1 | crest | present | 169 | 248 | 564 | 181 | 63 |
| 2 | crest | absent | 525 | 264 | 481 | 100 | 72 |
| 2 | crest | present | 462 | 1117 | 1472 | 105 | 95 |
| 3 | crest | absent | 1110 | 480 | 738 | 310 | 92 |
| 3 | crest | present | 467 | 657 | 715 | 103 | 22 |
| 4 | crest | absent | 912 | 292 | 599 | 288 | 76 |
| 4 | crest | present | 1034 | 345 | 891 | 422 | 72 |
| 5 | crest | absent | 324 | 387 | 240 | 318 | 63 |
| 5 | crest | present | 164 | 416 | 579 | 271 | 43 |
| 1 | footslope | absent | 1047 | 187 | 495 | 397 | 120 |
| 1 | footslope | present | 353 | 513 | 710 | 140 | 200 |
| 2 | footslope | absent | 756 | 244 | 722 | 234 | 147 |
| 2 | footslope | present | 52 | 392 | 380 | 185 | 163 |
| 3 | footslope | absent | 589 | 601 | 1759 | 167 | 87 |
| 3 | footslope | present | 708 | 1377 | 809 | 145 | 67 |
| 4 | footslope | absent | 272 | 215 | 771 | 202 | 40 |
| 4 | footslope | present | 13 | 291 | 388 | 202 | 64 |
| 5 | footslope | absent | 140 | 147 | 467 | 135 | 61 |
| 5 | footslope | present | 263 | 358 | 614 | 159 | 13 |
