# Supplementary material for: Change in Mesoherbivore Browsing Is Mediated by Elephant and Hillslope Position
Source: PLoS One. 2015 Jun 17;10(6):e0128340. doi: 10.1371/journal.pone.0128340 (PMC4471177; doi:10.1371/journal.pone.0128340)
Supplement: S5 Table — (DOCX) [file pone.0128340.s005.docx]

S5 Table. Soil properties per slope position in areas with and without elephant

| **Site number** | **Slope position** | **Elephant** | **P (mg/L)** | **K (mg/L)** | **Ca (mg/L)** | **Cu (mg/L)** | **Mg (mg/L)** | **Mn (mg/L)** | **Zn (mg/L)** | **Total cations (cmol/L)** | **pH (KCI)** | **Total % N** | **Sand (%)** | **Silt**  **(%)** | **Clay (%)** |
| --- | --- | --- | --- | --- | --- | --- | --- | --- | --- | --- | --- | --- | --- | --- | --- |
| 1 | crest | absent | 13 | 227 | 1063 | 5.9 | 212 | 9 | 1.2 | 7.65 | 5.56 | 0.15 | 68.98 | 17.27 | 13.73 |
| 1 | crest | present | 11 | 343 | 1079 | 5.1 | 210 | 15 | 1.0 | 8.02 | 6.13 | 0.07 | 73.60 | 13.03 | 9.51 |
| 2 | crest | absent | 17 | 249 | 1345 | 4.6 | 310 | 9 | 1.8 | 9.94 | 5.96 | 0.25 | 76.13 | 14.88 | 9.71 |
| 2 | crest | present | 17 | 192 | 1076 | 4.2 | 213 | 10 | 0.6 | 7.67 | 5.98 | 0.10 | 77.33 | 14.30 | 12.22 |
| 3 | crest | absent | 13 | 212 | 738 | 4.4 | 172 | 9 | 0.9 | 5.66 | 5.46 | 0.06 | 75.62 | 9.71 | 10.74 |
| 3 | crest | present | 17 | 263 | 923 | 4.9 | 191 | 11 | 0.9 | 6.88 | 5.57 | 0.07 | 77.42 | 14.85 | 9.07 |
| 4 | crest | absent | 14 | 298 | 1267 | 7.5 | 222 | 11 | 0.9 | 8.95 | 5.87 | 0.06 | 79.55 | 11.36 | 9.91 |
| 4 | crest | present | 10 | 266 | 871 | 7.0 | 233 | 11 | 1.6 | 6.99 | 5.86 | 0.11 | 74.68 | 13.46 | 11.18 |
| 5 | crest | absent | 12 | 184 | 698 | 6.5 | 146 | 14 | 1.3 | 5.21 | 5.39 | 0.11 | 78.77 | 11.58 | 8.68 |
| 5 | crest | present | 8 | 155 | 445 | 5.5 | 86 | 13 | 0.6 | 3.36 | 5.06 | 0.06 | 76.16 | 15.67 | 7.42 |
| 1 | footslope | absent | 20 | 207 | 1595 | 4.4 | 348 | 9 | 2.1 | 11.37 | 5.80 | 0.03 | 73.51 | 13.48 | 11.40 |
| 1 | footslope | present | 15 | 288 | 1542 | 4.1 | 315 | 7 | 1.1 | 11.05 | 5.85 | 0.11 | 76.57 | 12.37 | 10.72 |
| 2 | footslope | absent | 11 | 406 | 1953 | 5.0 | 343 | 8 | 1.8 | 13.63 | 6.22 | 0.10 | 78.27 | 9.35 | 9.97 |
| 2 | footslope | present | 17 | 317 | 1241 | 5.6 | 244 | 12 | 0.5 | 9.04 | 5.89 | 0.11 | 68.74 | 13.13 | 13.13 |
| 3 | footslope | absent | 28 | 279 | 1266 | 5.8 | 222 | 11 | 1.2 | 8.87 | 5.61 | 0.15 | 75.30 | 12.24 | 10.58 |
| 3 | footslope | present | 22 | 222 | 944 | 5.0 | 171 | 10 | 1.0 | 6.72 | 5.71 | 0.11 | 78.40 | 12.01 | 8.91 |
| 4 | footslope | absent | 8 | 220 | 584 | 4.3 | 131 | 14 | 0.4 | 4.62 | 5.38 | 0.24 | 78.58 | 13.00 | 9.08 |
| 4 | footslope | present | 10 | 219 | 887 | 5.5 | 187 | 12 | 0.8 | 6.55 | 5.59 | 0.07 | 80.78 | 15.25 | 10.51 |
| 5 | footslope | absent | 11 | 340 | 1732 | 12 | 325 | 8 | 0.8 | 12.21 | 5.65 | 0.04 | 74.90 | 12.17 | 8.67 |
| 5 | footslope | present | 12 | 585 | 1861 | 10 | 291 | 9 | 1.1 | 13.20 | 6.05 | 0.06 | 69.80 | 17.46 | 12.47 |
