# Supplementary material for: Change in Mesoherbivore Browsing Is Mediated by Elephant and Hillslope Position
Source: PLoS One. 2015 Jun 17;10(6):e0128340. doi: 10.1371/journal.pone.0128340 (PMC4471177; doi:10.1371/journal.pone.0128340)
Supplement: S6 Table — (DOCX) [file pone.0128340.s006.docx]

S6 Table. Browsing intensity (number of browsing events per hectare) for mesoherbivores and elephant per treatment (slope position and elephant presence/absence)

| **Site number** | **Slope position** | **Elephant** | **Mesoherbivore browsing** | **Elephant browsing** |
| --- | --- | --- | --- | --- |
| 1 | crest | absent | 62.50 | - |
| 1 | crest | present | 56.25 | 37.50 |
| 2 | crest | absent | 175.00 | - |
| 2 | crest | present | 466.67 | 21.61 |
| 3 | crest | absent | 200.00 | - |
| 3 | crest | present | 123.81 | 57.14 |
| 4 | crest | absent | 96.00 | - |
| 4 | crest | present | 269.23 | 12.09 |
| 5 | crest | absent | 20.95 | - |
| 5 | crest | present | 178.57 | 21.43 |
| 1 | footslope | absent | 65.00 | - |
| 1 | footslope | present | 48.52 | 40.00 |
| 2 | footslope | absent | 87.50 | - |
| 2 | footslope | present | 16.67 | 70.24 |
| 3 | footslope | absent | 37.25 | - |
| 3 | footslope | present | 141.67 | 60.12 |
| 4 | footslope | absent | 56.43 | - |
| 4 | footslope | present | 58.04 | 93.75 |
| 5 | footslope | absent | 55.00 | - |
| 5 | footslope | present | 243.75 | 31.25 |
